# Genome-Wide Identification of the Glycosyl Hydrolase Family 1 Genes in *Brassica napus* L. and Functional Characterization of *BnBGLU77*

**DOI:** 10.3390/plants14172686

**Published:** 2025-08-28

**Authors:** Xingzhi Wei, Yunshan Tang, Yuanyuan Liu, Shulin Shen, Jie Xu, Lulu Chen, Meifang Li, Huiyan Zhao, Ti Zhang, Hai Du, Huafang Wan, Cunmin Qu, Nengwen Yin

**Affiliations:** 1 Integrative Science Center of Germplasm Creation in Western China (Chongqing) Science City, College of Agronomy and Biotechnology, Southwest University, Chongqing 400715, China; weixingzhi031026@163.com (X.W.); tys1998@email.swu.edu.cn (Y.T.); lyy18865550726@163.com (Y.L.); ssl7159@email.swu.edu.cn (S.S.); 15723480213@163.com (J.X.); zxhcsgd1023@email.swu.edu.cn (L.C.); limf111@email.swu.edu.cn (M.L.); zhaohuiyan@swu.edu.cn (H.Z.); zhangti@swu.edu.cn (T.Z.); haidu81@126.com (H.D.); wanhua05@swu.edu.cn (H.W.); 2 Academy of Agricultural Sciences, Southwest University, Chongqing 400715, China; 3 Engineering Research Center of South Upland Agriculture, Ministry of Education, Chongqing 400715, China

**Keywords:** glycosyl hydrolase family 1 genes, *B. napus*, genome-wide analysis, catalytic function, stress adaptation

## Abstract

The β-glucosidases (BGLUs) of Glycoside Hydrolase Family 1 (GH1) exhibit essential functions in plant secondary metabolism and stress responses, mediated by their dual catalytic capabilities in hydrolysis and transglycosylation. This study identified 149 BGLU family members within *B. napus* (*Brassica napus* L.), which were systematically categorized into 10 distinct subgroups. Subsequent characterization encompassed detailed examination of their motif composition, chromosomal distribution, gene collinearity, selection pressure, and expression profiling. Transient overexpression of *BnBGLU77* in *N. benthamiana* (*Nicotiana benthamiana*), combined with untargeted metabolomics analysis, revealed pronounced modulatory effects on the degradation and accumulation of β-glucosidic compounds, suggesting potential roles of the protein encoded by *BnBGLU77* in metabolic homeostasis and stress response mechanisms. These experimental results first validated the bidirectional catalytic activity of a BGLU enzyme in *B. napus*, while simultaneously advancing fundamental understanding of *BnBGLU* gene functions and providing new insights for developing stress-resistant rapeseed cultivars through targeted genetic improvement.

## 1. Introduction

Amid escalating climate change, plants encounter diverse environmental challenges comprising both abiotic stressors (drought, salinity, temperature extremes) and biotic stresses (pathogenic infections) [1,2]. These stress factors significantly disrupt their growth and developmental processes, and lead to a decline in crop yields [3].The survival and developmental processes in plants critically rely on complex interplays between metabolic regulatory networks and sophisticated stress response mechanisms within the organism [4,5].

Among these regulatory processes, glycosylation modification, as a crucial biochemical regulatory means, plays an indispensable role [6]. Glycosyltransferases-mediated catalysis enables plants to attach diverse monosaccharide or oligosaccharide groups to substances such as small molecular compounds, proteins, and lipids, generating a huge and complex library of glycoside compounds [7]. These modified compounds play regulatory roles in developmental processes through hormone activity modulation and facilitation of substance translocation. Furthermore, glycosylated secondary metabolites contribute to stress adaptation by functioning as phytoalexins, modifying compound properties, and enhancing stress tolerance, thereby constituting essential defensive strategies against environmental challenges [8,9,10,11,12].

Glycoside hydrolases (EC 3.2.1), classified under glycosyl hydrolases (EC 3.2), represent a prominent subgroup of hydrolases (EC 3), among which glycoside hydrolase (GH) family 1 exhibits particularly notable β-glycosidase functionality [13,14]. As core members of the GH1 family, β-glucosidases (BGLUs) play a crucial role in regulating the dynamic balance of glycosylation. Leveraging their unique bidirectional catalytic activities of hydrolysis and transglycosylation, it has become an important molecular target for deciphering the mechanisms of plant development and environmental adaptation [15,16].

Structurally, BGLUs in the GH1 family possess highly conserved structural features, exhibiting a typical (α/β)_8_ TIM-barrel structure with a pocket-like catalytic channel [17]. BGLUs employ two catalytic pathways for hydrolysis: inversion and retention [17]. The inversion pathway proceeds through a single nucleophilic displacement, wherein the catalytic nucleophile of BGLU activates a water molecule through deprotonation. This nucleophilic water subsequently attacks the glycosidic bond to displace the aglycone and releases the sugar moiety with inversion of the configuration of the anomeric carbon. In contrast, the retention mechanism operates via a two-stage process. Firstly, the catalytic nucleophile attacks the anomeric carbon of the glucose residue, forming an enzyme–glucose intermediate. Second, a water molecule breaks this intermediate to release glucose. Usually, BGLU enzymes demonstrated transglycosylation activity under specific conditions, following an analogous mechanism where the second step involves nucleophilic attack by a sugar molecule instead of water, leading to the formation of a new glycosidic bond [17,18,19].

The functional diversity of BGLUs determines their critical roles in plant growth, development, and responses to environmental stresses. In *A. thaliana* (*Arabidopsis thaliana* (L.) Heynh.), the *AtBGLU26*-encoded enzyme exhibits broad-spectrum antifungal activity, while AtBGLU42 contributes significantly to systemic immunity against pathogens [20,21]. In rice (*Oryza sativa* L.), OsBGLU10, OsBGLU24, and OsBGLU33 participate in indole–3–acetic acid (IAA) and abscisic acid (ABA) signaling cascades [22], regulating physiological processes such as seed germination, root elongation, and drought tolerance [22]. Additionally, BGLU-mediated hydrolysis is crucial for activating dormant secondary metabolites and the process of cell wall synthesis [23]. Moreover, the transglycosylation activities of BGLUs, such as AtBGLU1, AtBGLU3, and AtBGLU4, could further expand the structural diversity of flavonoids, important specialized metabolites in plant-environment interactions [24,25]. Under stress conditions, the OsBGLU1 enzyme could mediate crosstalk between extracellular carbohydrate signaling and intracellular metabolic reprogramming through its structural features to catalyze transglycosylation of cell wall-derived oligosaccharides [15]. This enzymatic activity facilitates energy and carbon supply to maintain plant physiological functions [15,26].

*B. napus* (*Brassica napus* L., AACC genome), a globally significant oilseed crop, evolved through interspecific hybridization between *Brassica rapa* L.(AA) and *Brassica oleracea* L.(CC) accompanied by subsequent whole-genome duplication (WGD) [27]. This polyploidization facilitated widespread gene duplication events, generating multiple paralogous gene pairs that serve as genetic foundations for both subfunctionalization (partitioning ancestral functions among duplicates) and neofunctionalization (acquiring novel roles). While functional analyses of GH1 BGLUs have been conducted in various plant species, including *A. thaliana*, *O. sativa*, *Zea mays* L., *Pyrus bretschneideri* Rehder., *Fagopyrum tataricum* (L.) Gaertn., and *Medicago sativa* L. [14,20,21,22,23,28,29], the bidirectional catalytic activity and potential functions of BGLUs in *B. napus* remain uncharacterized. Consequently, systematic identification of the *BGLU* gene family members in *B. napus*, along with expression pattern analysis and catalytic function analysis, would establish a critical framework for elucidating the physiological functions of BGLUs in *B. napus*.

This study conducted a comprehensive genome-wide analysis of the *BGLU* gene family in *B. napus*, involving systematic identification of *BGLU* members and characterization of their conserved protein motif arrangements, gene structures, chromosomal localizations, and syntenic relationships. Additionally, we conducted expression pattern analysis and metabolomic profiling. The results obtained in this study lay a solid foundation for future in-depth research on *BGLU* genes in *B. napus*.

## 2. Results

### 2.1. Identification of BGLU Family Genes in B. napus

The conserved domain of the BGLU family (Pfam: PF00232) and the corresponding Hidden Markov Model (HMM) profile were retrieved from the Pfam database. Through integrated domain analysis, a total of 149 *BnBGLU* genes were systematically identified within the *BGLU* family. These genes were designated as *BnBGLU1* through *BnBGLU149* according to their chromosomal locations. Concurrently, their physicochemical properties, including protein size, molecular weight (MW), isoelectric point (pI), and predicted subcellular localization were conducted in this study (Table S1). Protein characterization revealed substantial structural diversity among BnBGLU family members. The encoded polypeptides exhibited length variation spanning 99–1147 amino acid residues, with an average of 460. Calculated molecular masses spanned from 11.81 to 128.68 kDa, with an average of 52.48 kDa. All isoelectric point (pI) values fell between 4.91 and 10.25, with the majority within the range of 5 to 9. Subcellular localization predictions indicated diverse compartmentalization patterns. The majority (87.25%) were located in the vacuole, while the rest were distributed across the endoplasmic reticulum, chloroplasts, cytoplasm, cell membrane, and nucleus.

### 2.2. Phylogenetic Analysis of BGLU Proteins in A. thaliana and B. napus

**Figure 1:**
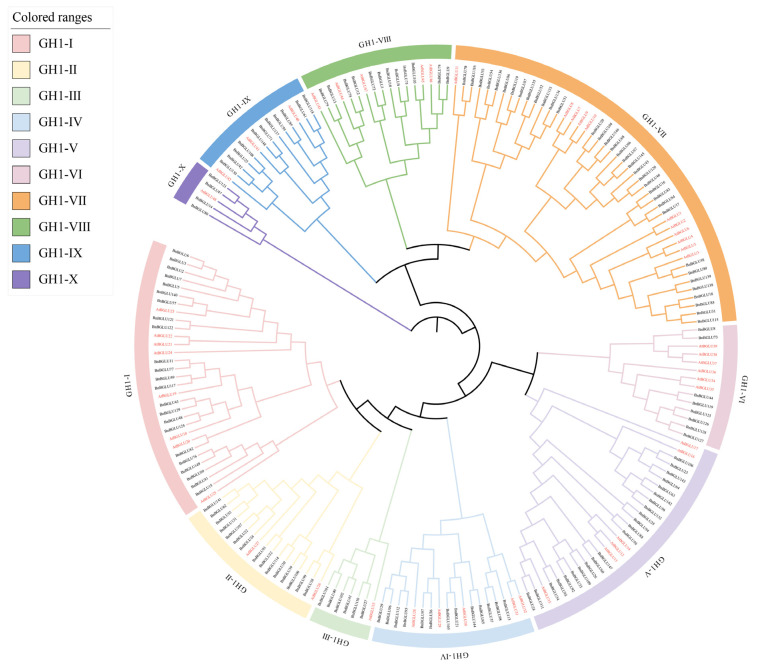
Phylogenetic tree depicting the relationships among the BGLU proteins from *A. thaliana* (*Arabidopsis thaliana* (L.) Heynh.) and *B. napus* (*Brassica napus* L.). The BGLU proteins were phylogenetically classified into ten subgroups, with the names of *A. thaliana* genes encoding these proteins labeled in red font.

In order to uncover the evolutionary relationship among the BnBGLU proteins in *A. thaliana* and *B. napus*, a phylogenetic analysis of both the BnBGLU and AtBGLU proteins was carried out. A rootless phylogenetic tree was generated to analyze the evolutionary relationships among 48 AtBGLU proteins and 149 BnBGLU proteins. The phylogenetic analysis classified all BGLU proteins into ten distinct subgroups, systematically labeled from GH1-I to GH1-X (Figure 1). Notably, the protein encoded by *AtBGLU48*, which is part of a separate lineage from the 10 subgroups in a previous study [30], was included in the GH1-X subgroup along with BnBGLU14, BnBGLU47, BnBGLU80, and BnBGLU123 proteins. The BnBGLU proteins were not evenly distributed across the ten subfamilies. Subfamily GH1-VII contained the most BnBGLU proteins, 35 in total, while subfamily GH1-III contained the fewest BnBGLU proteins, 6 in total. Overall, the number of BnBGLU proteins per subfamily varied, ranging from 6 to 35. Proteins within the same subfamily in *A. thaliana* have similar functions. Functional analyses have demonstrated that AtBGLU1 to AtBGLU6 contribute significantly to flavonoid biosynthesis in *A. thaliana* [31], while AtBGLU45, AtBGLU46 and AtBGLU47 participate in lignin biosynthesis pathway [32,33]. It can reasonably be inferred that the proteins encoded by these homologous genes in *B. napus* could putatively perform similar functions.

### 2.3. Analysis of Conserved Motifs and Gene Structure of the BnBGLU Family

**Figure 2:**
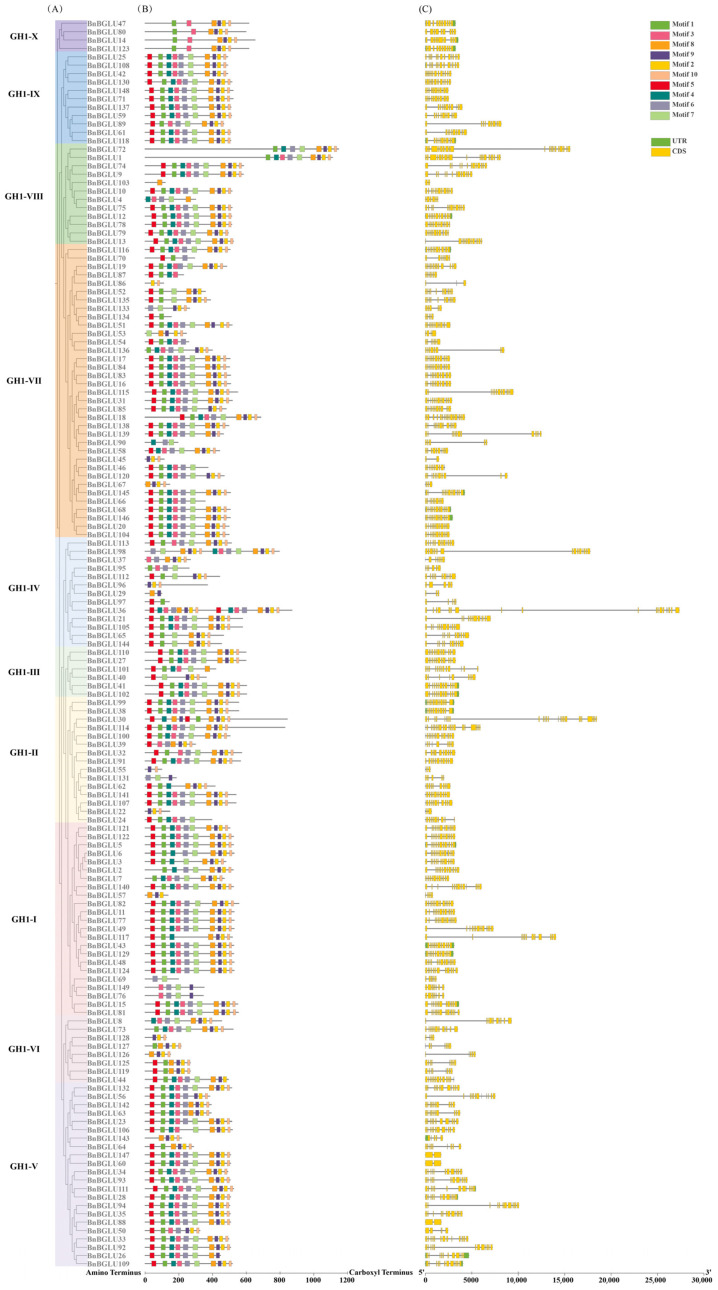
Integrated analysis of BGLU protein phylogeny, conserved motifs, and encoding gene structures in *B. napus*. (**A**) Phylogenetic tree depicting the relationships of the BnBGLU proteins. (**B**) Conserved motifs of BnBGLU proteins: ten motifs were identified using MEME and are represented by different colors, respectively. (**C**) Gene structure of *BnBGLU* genes. The yellow boxes represent the coding sequences (CDS), the green boxes denote the untranslated regions (UTRs), and the solid gray lines indicate the introns.

To further understand the relationships within the BGLU protein family, we constructed a combined diagram of the phylogenetic tree, motif patterns and the structure of the genes encoding these proteins (Figure 2). MEME software (version 5.5.8) analysis identified ten conserved motifs in the BGLU proteins, sequentially designated as motif 1 to motif 10. Among them, motif 7 stood out as the longest one, containing 30 amino acid residues. Conversely, the shortest was motif 10, with only 16 amino acid residues (Table S2). Notably, BnBGLU36 and BnBGLU98, displayed duplicated motifs, whereas several members retained only 1–3 conserved motifs. Comprehensive analysis of BGLU protein motif distribution revealed subgroup-specific architectural patterns. While motif variation existed across phylogenetic clades, proteins within individual subgroups maintained conserved motif arrangements.

Previous studies have shown that the catalytic activity centers of GH1 family BGLUs generally involve two highly conserved motifs: the ITENG motif and the TFNEP motif [34]. In our analysis of BnBGLU proteins, we found that motif 8 harbors a complete ITENG sequence. Meanwhile, motif 3 has a TINE sequence, which may be a homologous motif of TFNEP (Table S2). Other homologous motifs TINQL and TLNEP were also discovered in *Sinapis alba* and *F. tataricum* [23,35], demonstrating the inherent motif plasticity among plant GH1 enzymes. Among the 149 BnBGLU proteins analyzed, 121 members (81.2%) harbor motif 8, while 112 members (75.2%) possess motif 3. Furthermore, 100 members (67.1%) contain both motifs.

The visual analysis of exon/intron structures showed a substantial structural diversity among *BnBGLU* genes, spanning from 2 to 24 exons per gene. The majority of these genes exhibited conserved exon counts between 10 and 13, reflecting evolutionary conservation of these structural configurations. Notably, five genes (*BnBGLU45*, *BnBGLU60*, *BnBGLU88*, *BnBGLU103*, and *BnBGLU147*) exhibited minimal structural complexity with merely two exons, while, *BnBGLU30* displayed the most complex architecture with 24 exons. Phylogenetic subgroup analysis revealed conserved exon–intron patterns among evolutionarily related members, with major structural divergence primarily occurring between different clades. This conservation pattern correlated strongly with phylogenetic relationships, suggesting parallel evolutionary trajectories within subgroups. Notably, the correlation between phylogenetic proximity and structural similarity further supports the potential role of exon–intron organization may serve as a conserved feature in gene family evolution.

### 2.4. Chromosome Mapping Analysis of BnBGLU Genes

**Figure 3:**
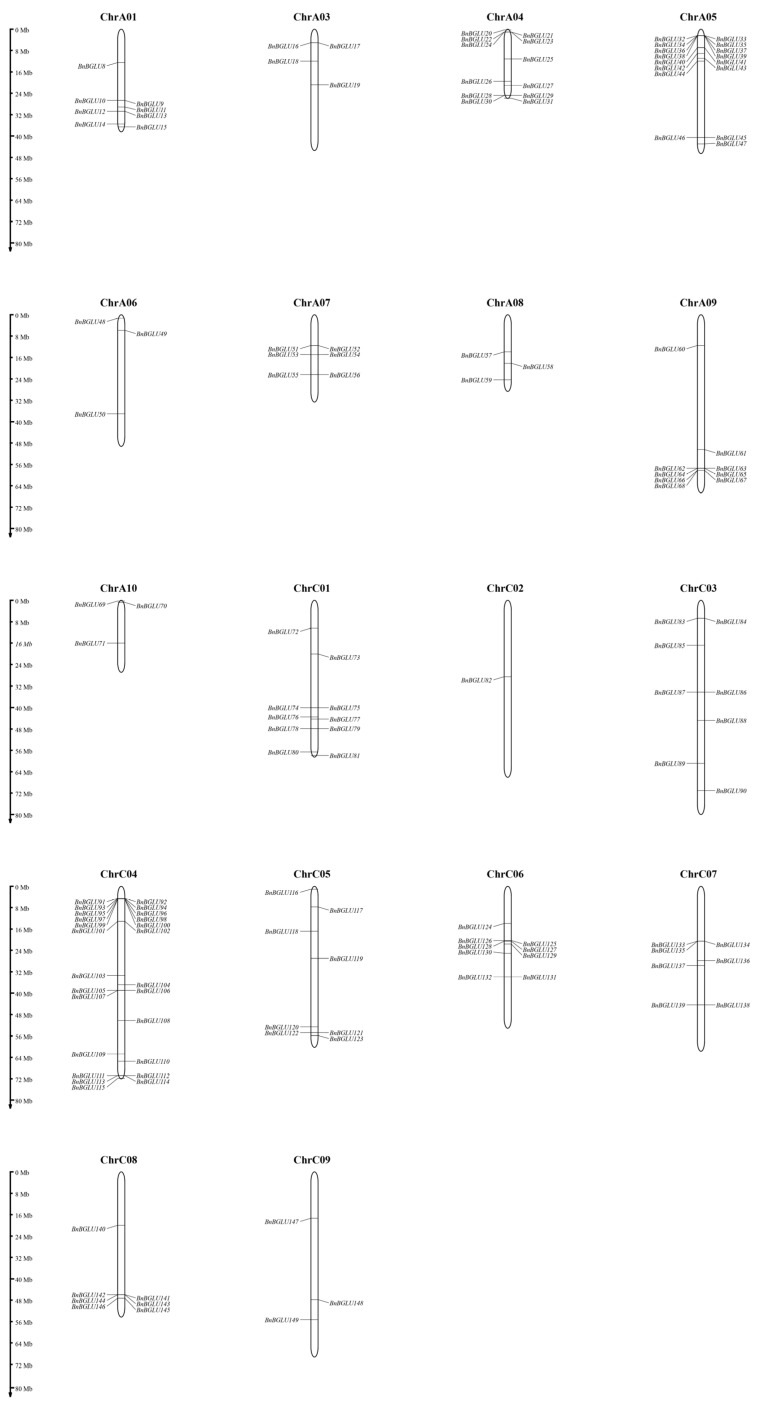
Chromosomal localization of *BGLU* genes in *B. napus*. Vertical bars represent the chromosomes of *B. napus*. Above each chromosome is the corresponding chromosome number, and the scale on the left indicates the chromosome length.

Chromosomal localization analysis identified an uneven distribution pattern of *BnBGLU* genes across 18 chromosomes, excluding seven genes (*BnBGLU1-7*) located on unassembled scaffold regions (Figure 3). Among the 142 mapped genes, chromosome C04 displayed maximal gene density with 25 members representing six phylogenetic subgroups: seven genes from subgroup IV, six genes from subgroup V, five genes from subgroup II, three genes from subgroup III, two genes from subgroup VII, and one gene each from subgroups VIII and IX. However, chromosome C02 contained merely one subgroup I gene. The absence of discernible distribution patterns across subgroups suggests stochastic chromosomal distribution patterns, possibly shaped by differential selective pressures during genome evolution.

### 2.5. Collinearity and Selection Pressure Analysis of BGLU Genes

**Figure 4:**
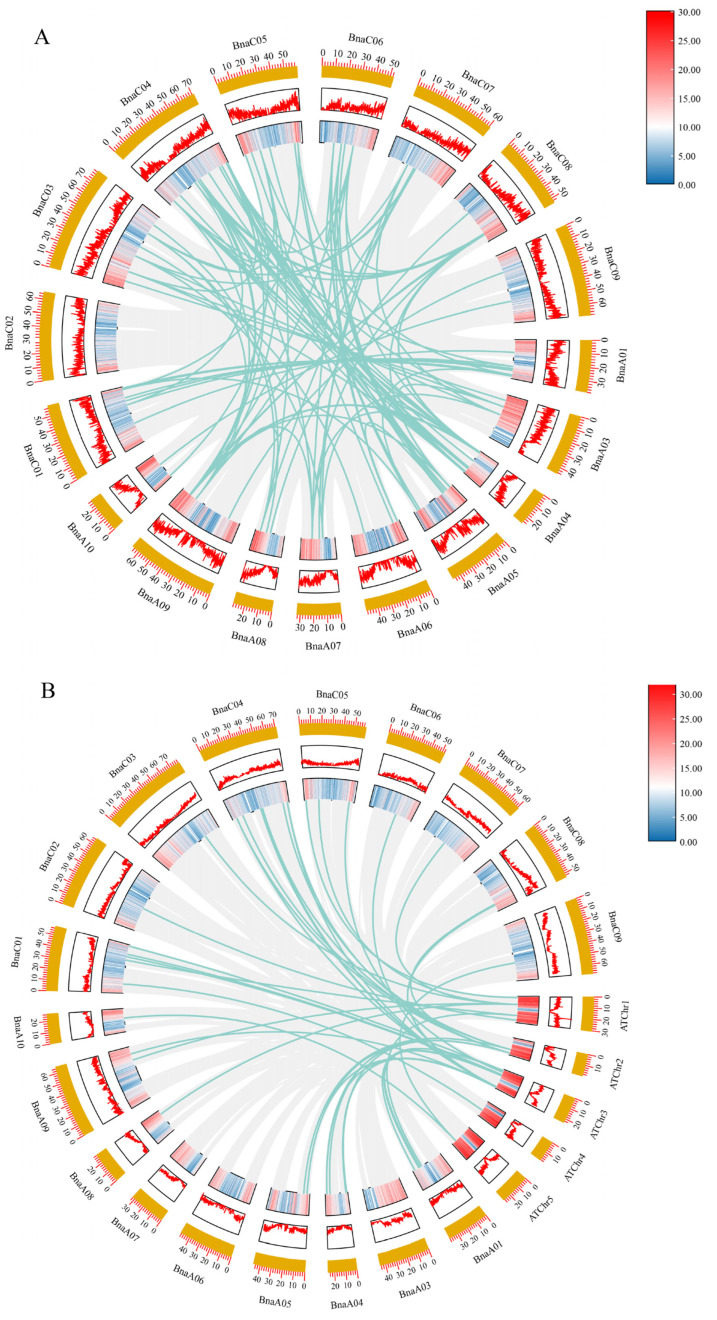
Collinearity analysis of *BGLU* family genes between *A. thaliana* and *B. napus*. (**A**) The collinearity analysis within *B. napus*. (**B**) The collinearity analysis between *A. thaliana* and *B. napus*. The gray lines in the background represent the collinear blocks within *B. napus* or between *A. thaliana* and *B. napus*, and the blue lines denote the collinear *BGLU* gene pairs. The orange blocks represent the chromosomes. Inside the chromosomes, gene density is presented in the form of a heatmap and a line graph, respectively.

Collinearity analysis of *BnBGLU* gene family members in *B. napus* revealed 155 conserved homologous gene pairs, providing compelling evidence that gene duplication serves as the primary mechanism driving *BGLU* family expansion in this polyploid species [36]. Additionally, systematic comparative analysis revealed 60 collinear orthologous relationships between *A. thaliana* and *B. napus*, comprising 19 *AtBGLU* genes and 50 *BnBGLU* genes. The conservation of these evolutionarily conserved gene pairs suggests preservation of core biochemical functions and transcriptional regulatory mechanisms [37], simultaneously revealing both lineage-specific diversification patterns and functional conservation of *BGLU* genes across Brassicaceae species (Figure 4, Tables S3 and S4).

To investigate evolutionary patterns of chromosomal duplication events, comprehensive Ka/Ks ratio analysis was performed (Tables S3 and S4). Notably, the *BnBGLU51/BnBGLU133* gene pair exhibited a Ka/Ks ratio of 1.87, indicating strong positive selection effect acting on this particular gene pair, signifying that beneficial mutations have been preferentially retained during the course of evolution [38]. In contrast, the Ka/Ks values of the remaining homologous *BnBGLU* gene pairs, along with those of the *AtBGLU-BnBGLU* homologous gene pairs, were all found to be less than 1. These findings demonstrate clear purifying selection acting on homologous *BGLU* gene family pairs throughout *B. napus* evolution, a process that removes detrimental mutations while preserving gene family integrity and functionality [38,39]. Furthermore, three specific gene pairs (*BnBGLU22/BnBGLU39*, *BnBGLU29/BnBGLU113*, and *BnBGLU38/BnBGLU62)* exhibited complete absence of synonymous substitution (Ks), likely resulting from an extraordinary sequence conservation under intense purifying selection pressure that effectively constrained synonymous nucleotide substitutions in coding regions to near-undetectable levels [40].

### 2.6. Prediction of Cis-Acting Elements in the BnBGLU Promoter Regions

**Figure 5:**
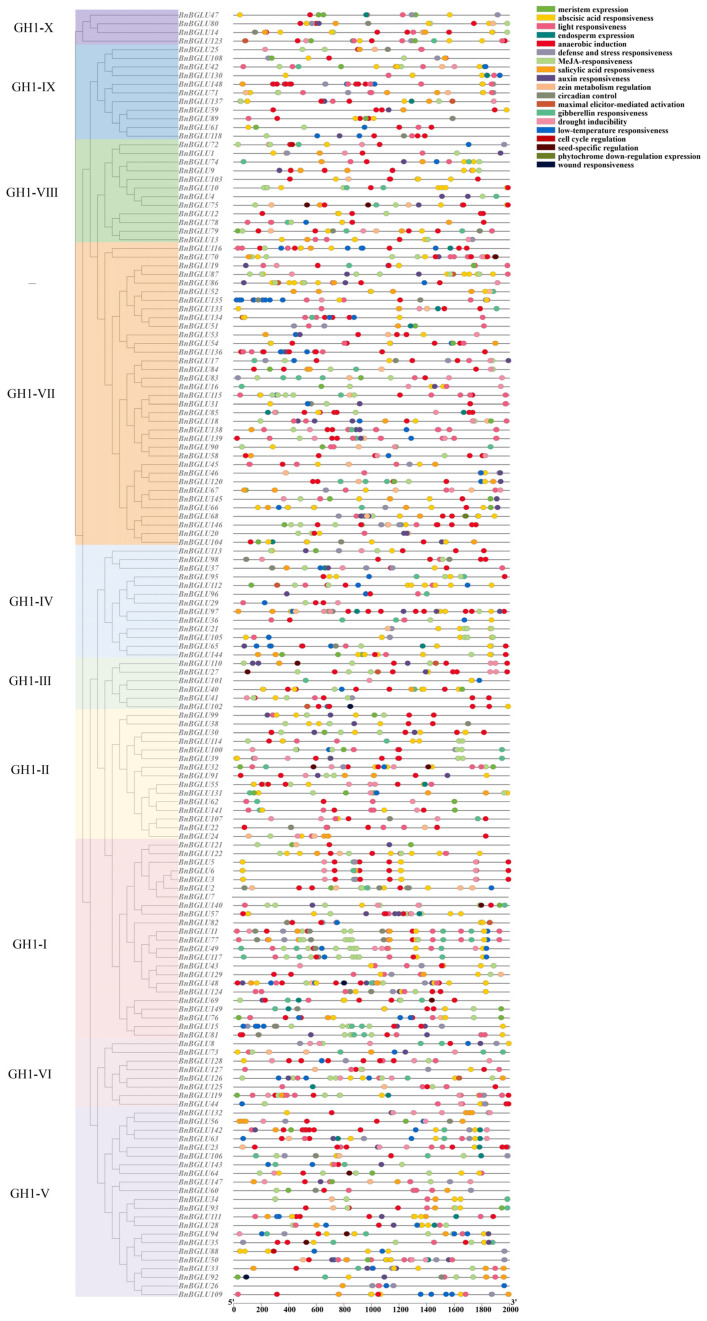
Predicted cis-acting elements in the promoters of *BnBGLUs*.

Putative cis-regulatory elements within the 2000 bp promoter regions upstream of 149 annotated *BnBGLU* genes were predicted using PlantCARE software (version 1) (Figure 5). The analysis predicted a functionally diverse array of cis-regulatory elements involved in three key biological processes: cellular proliferation, developmental programming, and stress adaptation mechanisms. The transcriptional regulation of *BnBGLU* genes integrates multiple phytohormone signaling pathways, as evidenced by the presence of response elements for abscisic acid, methyl jasmonate, and auxin. Environmental stress-activated regulatory nodes, including those mediating anaerobic induction, drought induction, and low-temperature adaptation, reveal sophisticated transcriptional control mechanisms that enable *B. napus* to maintain metabolic homeostasis despite environmental fluctuations through coordinated gene expression adjustments [41]. Developmental cis-elements associated with cell cycle regulation and meristematic activity further implicate *BnBGLUs* in spatiotemporal coordination of organogenesis and tissue differentiation, reflecting their evolutionary conservation in developmental plasticity [42]. These results demonstrate the essential regulatory functions of *BnBGLU* genes in *B. napus* development and stress adaptation, revealing molecular mechanisms underlying their functional diversification and adaptive strategies.

### 2.7. Expression Analysis of BnBGLU Genes in B. napus Under Phosphorus and Phytohormone Treatment

**Figure 6:**
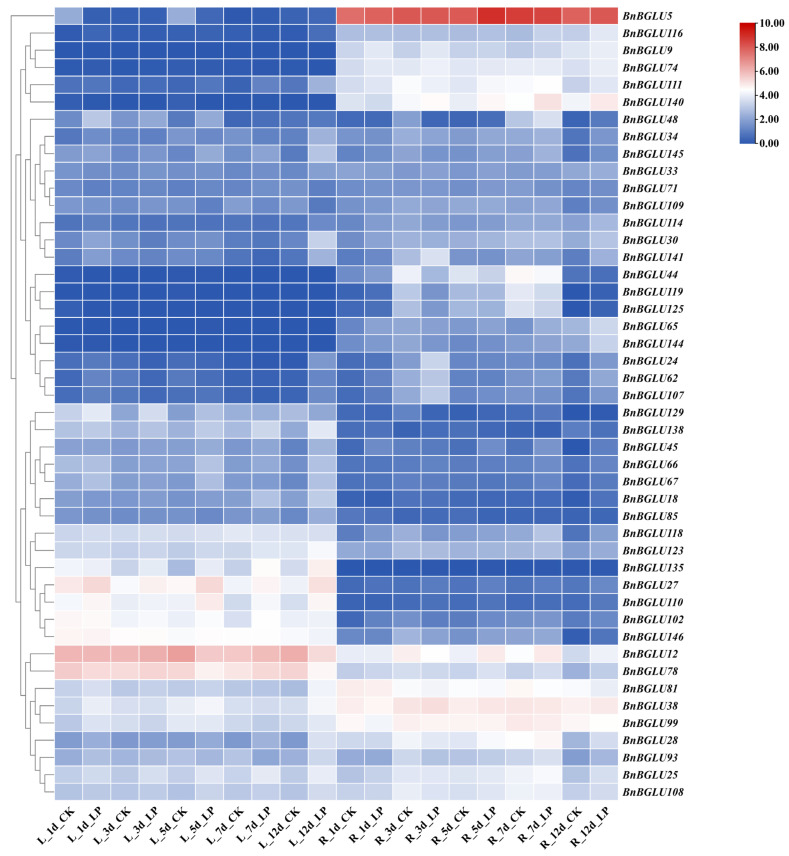
Expression profiles of *BnBGLU* genes under phosphorus treatments. Data were normalized using Log_2_ (FPKM + 1) values. Samples include L (leaf) and R (root), with time points indicated as d (days post treatment). Experimental conditions: LP (low-phosphorus treatment) and CK (control, nutrient solution without phosphorus deficiency). FPKM: fragments per kilobase of exon model per million mapped fragments.

**Figure 7:**
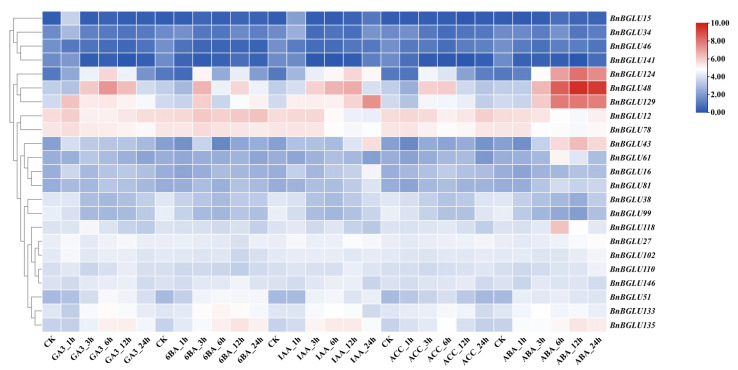
Expression profiles of *BnBGLU* genes under phytohormone treatments. Data were normalized using Log_2_ (FPKM + 1) values. Experimental conditions: CK (control, nutrient solution without phytohormone treatment), GA_3_ (gibberellic acid), 6-BA (6-benzyladenine), IAA (indole-3-acetic acid), ACC (1-aminocyclopropane-1-carboxylic acid), and ABA (abscisic acid). Time points are indicated as h (hours post-treatment). FPKM: fragments per kilobase of exon model per million mapped fragments.

As a critical mineral element for plant physiology, phosphorus (P) deficiency induces root system architecture remodeling and activates P-signaling transduction cascades [43]. Previous studies have established the upregulation of *BGLU* genes during P-deprivation stress, where these genes contribute to P recycling, root morphological modifications, and stress adaptation [44,45,46]. Moreover, phytohormone networks function as pivotal regulators that integrate stress responses, with growth modulation and environmental acclimation [47]. To elucidate *BnBGLUs* gene functions, expression patterns were examined under both low-phosphorus (LP) stress and phytohormone treatments (Figure 6 and Figure 7).

The results revealed distinct tissue-specific expression patterns among *BnBGLU* family members. Under both normal and LP conditions, the majority of genes displayed tissue-specific expression patterns with preferential accumulation in either roots or leaves, while a limited subset demonstrated constitutive expression across both organs. LP treatment triggered significant transcriptional reprogramming of multiple *BnBGLU* genes in these organs, suggesting their potential roles in P stress adaptation. Root tissues exhibited distinct temporal expression profiles: *BnBGLU12*, *BnBGLU28*, and *BnBGLU140* showed progressive upregulation throughout the LP treatment, while *BnBGLU24*, *BnBGLU62*, and *BnBGLU107* displayed transient induction, reaching maximal expression at 3 days before subsequent attenuation. Conversely, *BnBGLU44*, *BnBGLU119*, and *BnBGLU125* were consistently downregulated at both mid-term (3-day) and extended (7-day) treatment stages. In leaf tissues, LP stress markedly elevated transcript levels of *BnBGLU25*, *BnBGLU27*, *BnBGLU102*, *BnBGLU110*, and *BnBGLU135*. Temporal stratification was also evident: early-responsive genes *BnBGLU48* and *BnBGLU129* maintained elevated expression levels throughout the initial 5-day treatment phase, while delayed-response members such as *BnBGLU30* displayed significant activation only after prolonged stress (12 days). Notably, *BnBGLU5* demonstrated a biphasic repression pattern, with marked downregulation observed at both 1-day and 5-day intervals. These coordinated expression dynamics reveal sophisticated regulatory networks that fine-tune *BnBGLU* gene activity in a temporal and organ-specific manner to accommodate physiological requirements during phosphorus limitation (Figure 6).

Distinct regulatory patterns emerged from the expression dynamics of *BnBGLU* genes under treatment with five phytohormones (GA_3_, 6-BA, IAA, ACC, ABA) (Figure 7). Three genes (*BnBGLU48*, *BnBGLU124*, and *BnBGLU129)* showed substantial transcriptional modulation in response to all tested hormones, implying their potential roles as integrative hubs in hormonal cross-talk. In contrast, another gene set comprising *BnBGLU12*, *BnBGLU38*, *BnBGLU78*, and *BnBGLU99* displayed oscillatory expression profiles with an overall downward trend, possibly indicating feedback attenuation or hormone-induced metabolic shifts. Particular genes demonstrated selective hormone responsiveness: *BnBGLU43* displayed significant upregulation specifically in response to IAA and ABA, whereas *BnBGLU61* and *BnBGLU118* exhibited ABA hypersensitivity, both attaining maximal expression at 6 h post-ABA treatment. These differential expression patterns implicate *BnBGLU* genes in the sophisticated integration of phytohormone signals that potentially coordinate growth and stress adaptation in *B. napus*.

### 2.8. Tissue Expression Analysis of BnBGLU Genes

**Figure 8:**
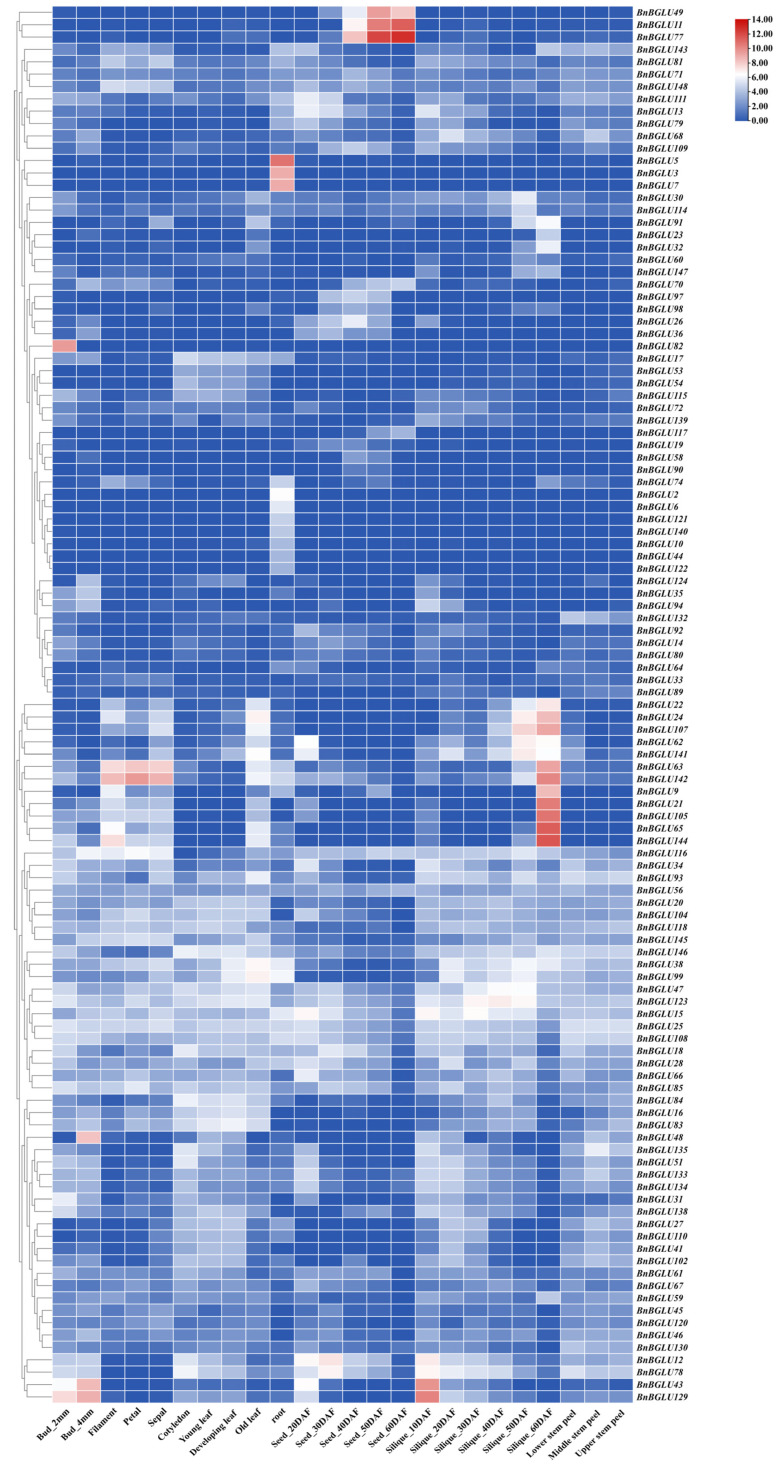
Expression profiles of *BnBGLU* genes in diverse tissues and organs. Data were normalized using Log_2_ (FPKM + 1) values. 2 mm and 4 mm denote bud diameters of 2 or 4 mm, respectively. DAF: days after flowering. FPKM: fragments per kilobase of exon model per million mapped fragments.

To further elucidate the functional contributions of *BnBGLU* genes during *B. napus* development, a systematic investigation of their expression patterns was conducted across diverse tissue types, developmental stages, and spatial positions within the same tissue (Figure 8). The analysis revealed predominant involvement of *BnBGLU* genes in developmental regulation, with distinct spatiotemporal specificity in their expression patterns. For instance, specific members including *BnBGLU3*, *BnBGLU5*, and *BnBGLU7* showed root-specific expression, whereas *BnBGLU11*, *BnBGLU49*, and *BnBGLU77* displayed significantly elevated expression levels in seeds at 40, 50, and 60 days post anthesis. Notably, partial genes displaying expression patterns similar to *BnBGLU144* exhibited marked upregulation in late silique developmental stages, while other members including *BnBGLU129* reached maximal expression levels during initial silique development followed by progressive downregulation. These genes with opposing expression patterns likely coordinate silique development through stage-specific functional contributions. These observations collectively demonstrate the existence of divergent regulatory strategies within the *BGLU* family throughout *B. napus* development, highlighting their critical roles in both vegetative growth and reproductive organogenesis.

### 2.9. The Effect of Transient Expression of BnBGLU77 on Metabolites in N. benthamiana (Nicotiana benthamiana)

**Figure 9:**
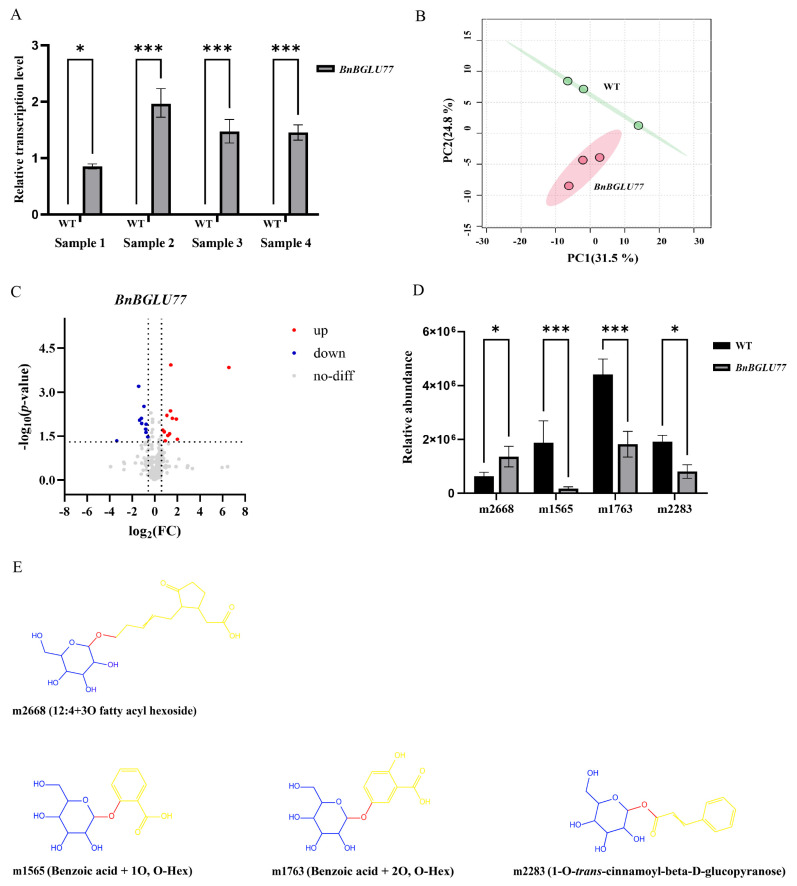
Untargeted metabolomic analysis of *BnBGLU77*: (**A**) qRT-PCR analysis of *BnBGLU77* expression levels in *N. benthamiana* (*Nicotiana benthamiana*). Gray bars represent relative transcriptional levels of *BnBGLU77* in *BnBGLU77*-overexpressing *N. benthamiana* leaves. Samples 1–4 correspond to *N. benthamiana* leaves subjected to transient expression experiments. Statistical significance: * *p* < 0.05, *** *p* < 0.001. (**B**) PCA of metabolomic data from *N. benthamiana* leaves. PC1 explains 31.5% of variance, while PC2 accounts for 24.8%. (**C**) Volcano plot analysis of differential metabolites. Metabolites were classified based on the criteria of VIP > 1, |log_2_(fold change)| > 0.585, and *p* < 0.05: red dots denote significantly upregulated metabolites, blue dots represent significantly downregulated metabolites, and gray dots indicate metabolites with no significant alteration. Dashed lines demarcate the thresholds for |log_2_(fold change)| > 0.585 (horizontal) and −log_10_(*p*) ≥ 1.3 (vertical), corresponding to the cutoff of *p* < 0.05. (**D**) Relative abundance analysis of significantly altered target metabolites. Black and gray bars illustrate metabolite relative levels in controls and *BnBGLU77*-overexpressing *N. benthamiana* leaves, respectively. Statistical significance: * *p* < 0.05, *** *p* < 0.001. (**E**) Chemical structures of identified target metabolites (one significantly upregulated and three significantly downregulated). Key structural features are annotated using blue for glucosyl moieties, red for β-glucosidic bonds, and yellow for aglycone moieties.

Considering the status of seeds as the principal harvested organ in *B. napus*, we selected the seed-specific gene *BnBGLU77*, based on tissue expression transcriptome analysis (Section 2.8), for functional validation. To elucidate its function, we conducted transient expression assays in *N. benthamiana*. qRT-PCR analysis revealed significantly elevated expression levels in samples. This indicates successful overexpression of the gene in these leaf samples (Figure 9A). Subsequently, we selected Sample 2, which exhibited the highest expression level, for untargeted metabolomic analysis.

Principal component analysis (PCA) revealed clear metabolic divergence between transgenic samples and control groups, indicative of BnBGLU77 protein-induced metabolic alteration (Figure 9B). Differential metabolites identification employed a multi-criteria approach incorporating: (1) variable importance in projection (VIP) threshold > 1 derived from the orthogonal partial least squares-discriminant analysis (OPLS-DA) model, (2) independent filter of |log_2_(fold change)| > 0.585, and (3) statistical significance threshold of *p* < 0.05. Finally, we identified 12 metabolites with significant upregulation and 10 with downregulation, all potentially involved in BnBGLU77 protein-mediated pathway regulation (Figure 9C). Notably, structural characterization of this subset identified four metabolites harboring β-glucosidic bonds, including three downregulated species, benzoic acid + 1O (m1565), benzoic acid + 2O (m1763), 1-O-*trans*-cinnamoyl-beta-D-glucopyranose (m2283); and a singular upregulated metabolite, 12:4 + 3O fatty acyl hexoside (m2668) (Figure 9D,E). The observed results demonstrate consistency with the bidirectional catalytic activities (hydrolysis and transglycosylation) of BGLUs, with hydrolysis of β-glucosidic bonds representing the predominant catalytic activity [48]. Furthermore, metabolite m2668 has been documented to contribute substantially to enhanced plant pathogen defense mechanisms [49,50]. Concurrently, the results showed that β-glucosidase-mediated hydrolysis of metabolites m1565, m1763, and m2283 generated salicylic acid (2-hydroxybenzoic acid), gentisic acid (2,5-dihydroxybenzoic acid), and *trans*-cinnamic acid ((E)-3-phenylprop-2-enoic acid) as their respective hydrolytic products. These phenolic compounds are well-characterized mediators of plant stress adaptation responses [51,52,53,54,55,56,57,58,59,60].

## 3. Discussion

GH1 BGLUs represent a class of multifunctional enzymes critical for plant environmental adaptation. These enzymes possess dual catalytic capabilities, namely hydrolysis and transglycosylation, which facilitate dynamic modulation of glycosylation states and subsequent regulation of plant growth, defense, and stress resilience [15,16,20,21,22,23,24,25,26]. This study identified 149 *BnBGLU* genes in *B. napus*, substantially more than the 48 members in its diploid relative *A. thaliana*, suggesting that polyploidization events primarily contributed to this gene family expansion. Phylogenetic classification divided the BGLU proteins into 10 subgroups (GH1-I to GH1-X), with categorization patterns highly similar to those of functionally diversified BGLU proteins in *A. thaliana* (Figure 1) [34]. Notably, asymmetric subgroup distribution was observed, as exemplified by GH1-VII housing 35 members, suggesting subgroup-specific expansion within the *BnBGLU* family (Figure 1). Collinearity analysis revealed 155 segmental duplication gene pairs as the principal mechanism underlying gene family expansion (Table S3), a finding that consistent with the evolutionary strategy of polyploid species leveraging genetic redundancy to enhance functional plasticity [36]. The prevalence of purifying selection (Ka/Ks < 1) across most paralogs underscores evolutionary conservation of core enzymatic functions within this family (Tables S3 and S4) [38,39].

Structural analyses indicated substantial divergence among BnBGLU proteins, including variation in length, physicochemical diversity, and subcellular localization, which reflects functional specialization shaped by evolutionary pressures (Table S1). The vacuolar-localized BnBGLU proteins likely participate in glycoside hydrolysis, potentially contributing to three key physiological processes: plant defense, secondary metabolite biosynthesis, and nutrient mobilization [61]. Additionally, the widespread subcellular distribution of BnBGLU proteins across additional compartments further implies functional versatility. Comparative analysis of BnBGLU protein architectures revealed evolutionary patterns underlying functional diversification (Figure 2). Subgroup-specific conservation patterns of structural motifs point to selective maintenance of critical structural elements during the evolution of the BnBGLU protein family. The catalytic activity of GH1 family BGLUs specifically depends on two highly conserved glutamate residues: a nucleophilic glutamate embedded within the ITENG motif and an acid/base catalytic glutamate typically situated in the TFNEP motif. These signature peptide motifs form the catalytic core facilitating both glycosylation and deglycosylation reactions [34]. Notably, most BnBGLU members possess both motif 3 and motif 8, indicating that the majority of this family likely retains catalytic activity. Remarkably, the two proteins, BnBGLU36 and BnBGLU98 exhibit duplicated motifs that may confer selective advantages through functional enhancement or neofunctionalization [62]. The reduction in motifs observed in certain protein members probably represents adaptive modifications in response to distinct functional requirements or environmental constraints [63]. Concurrently, exon–intron structural conservation within phylogenetic subgroups indicated evolutionary trajectory sharing among related members [64]. The significant correlation between architectural complexity and phylogenetic clustering implies that both motif configurations and gene structures collectively serve as conserved evolutionary markers.

Promoter analysis revealed putative cis-elements linked to growth regulation, stress responses, and hormonal signaling pathways (Figure 5), revealing the sophisticated transcriptional control of *BnBGLUs*. The spatiotemporal expression dynamics of *BnBGLU* genes under P deprivation revealed hierarchical regulatory mechanisms mediating stress adaptation in *B. napus* (Figure 6). The observed tissue-specific activation or repression patterns and temporally stratified expression kinetics reflect functional diversification among family members. Such differential regulation appears to align with stage-specific physiological demands, where rapidly induced genes mediate initial stress signaling while delayed responders facilitate long-term acclimation. These findings collectively identify *BnBGLU* genes as crucial coordinators bridging immediate stress detection with sustained metabolic adaptation under P restriction. Furthermore, the heterogeneous expression patterns of *BnBGLU* genes under the treatment of various phytohormones implicates these proteins as nodal points integrating hormonal cues to balance growth processes with environmental adaptation (Figure 7).

Tissue-specific expression analysis of *BnBGLU* genes demonstrated broad functional involvement in *B. napus* growth and developmental regulation (Figure 8). The seed-specific expression pattern of *BnBGLU77* prompted its selection for targeted metabolomic characterization (Figure 9). Metabolite profiling detected substantial accumulation of β-glucosidic compounds classified as fatty acid acyl glycosides, compounds previously established as broad-spectrum antimicrobial agents contributing to pathogen resistance [49,50]. Notably, metabolites benzoic acid + 1O, benzoic acid + 2O, and 1-O-*trans*-cinnamoyl-beta-D-glucopyranose exhibited significant downregulation following β-glucosidase hydrolysis, yielding salicylic acid (2-hydroxybenzoic acid), gentisic acid (2,5-dihydroxybenzoic acid), and *trans*-cinnamic acid ((E)-3-phenyl-2-propenoic acid), respectively. These defense-related compounds exhibit well-characterized roles in plant protection mechanisms. Salicylic acid (SA) functions as a central signaling molecule that coordinates stress responses and interacts synergistically with various phytohormones, including abscisic acid (ABA) and ethylene [51,52]. The SA derivative gentisic acid similarly participates in plant-pathogen defense and stress responses [10,53,54]. *Trans*-cinnamic acid contributes to defense by promoting SA accumulation and phenolic biosynthesis, while simultaneously serving as a pivotal precursor for diverse secondary metabolites involved in plant defense and allelopathy [55,56,57,58,59,60]. Collectively, the coordinated action of these metabolites potentially enhances *B. napus* stress tolerance by regulating interconnected biochemical pathways responsible for synthesizing diverse defense compounds.

This study performed comprehensive genome-wide identification and expression profiling of the *BnBGLU* genes in *B. napus*. Metabolomic analysis revealed the bidirectional catalytic activities of BnBGLU77 enzyme, encompassing both hydrolytic cleavage and transglycosylation, while underscoring the potential roles of BnBGLU77 protein in environmental stress adaptation. These findings establish a foundation for elucidating the biological functions of BnBGLU proteins and provide valuable insights for developing stress-resistant *B. napus* cultivars through targeted genetic improvement.

## 4. Materials and Methods

### 4.1. Identification and Analysis of BnBGLU Genes

In this study, protein sequences of *A. thaliana* BGLUs were retrieved from the TAIR database (https://www.arabidopsis.org/ (accessed on 11 November 2024)) [65], while the protein sequences of *B. napus* (ZS11.v0) were obtained from the *B. napus* Multi-Omics Information Resource (BnIR, https://yanglab.hzau.edu.cn/BnIR (accessed on 11 November 2024)) [66]. Initially, the conserved domain of the BGLU family (Pfam: PF00232) was identified using the Pfam database (version 37.4; http://pfam.xfam.org/ (accessed on 11 November 2024)) [67], and the corresponding hidden Markov model (HMM) profile was downloaded from the database. Candidate genes were screened from the *B. napus* ZS11 genome with an HMM threshold E-value < 1 × 10^−10^. Subsequently, homologs genes of the 48 known *A. thaliana BGLU* genes were identified using TBtools-II (version 2.315)-Quick Find Best Homology and the *B. napus* Multi-Omics Information Resource (BnIR, https://yanglab.hzau.edu.cn/BnIR (accessed on 11 November 2024)) [66,68]. Domain analysis was then performed using NCBI CD-search (https://www.ncbi.nlm.nih.gov/Structure/cdd/wrpsb.cgi (accessed on 11 November 2024)) to confirm the presence of the Glycosyl Hydrolase family 1 domain [69]. Members devoid of the essential functional domain were omitted from the analysis. Ultimately, 149 *BnBGLU* genes within the *BGLU* family were identified. Protein sequence parameters, including length, molecular weight (MW in kDa), and isoelectric point (pI), were predicted using TBtools-II (version 2.315) “Protein Parameter Calc” module [68]. Subcellular localization predictions were performed using Plant-mPLoc 2.0 (http://www.csbio.sjtu.edu.cn/bioinf/plant-multi/ (accessed on 17 November 2024)) [70].

### 4.2. Phylogenetic Analysis of the BGLU Family Members

A maximum likelihood (ML) phylogenetic tree was built using TBtools-II (version 2.315) “One Step Build a ML Tree” module with default parameters [68]. The resulting tree was subsequently visualized and annotated using Interactive Tree of Life (iTOL) platform (version 7.0; https://itol.embl.de (accessed on 15 January 2025)) [71].

### 4.3. Conserved Motif Identification and Gene Structure Analysis of the BnBGLU Family

Conserved motifs within BGLU family members were identified and analyzed using the Multiple Expectation Maximization for Motif Elicitation (MEME) tool (version 5.5.8; https://meme-suite.org/meme/ (accessed on 25 January 2025)) with parameters: number of repetitions (arbitrary), maximum number of motifs (10), and width of motifs (6-300 amino acid residues) [72]. Gene exon–intron structures, motif patterns, and phylogenetic relationships were integrated and visualized using the gene structure visualization function in TBtools-II (version 2.315) [68].

### 4.4. Analysis of the Chromosomal Localization of BnBGLU Genes

Chromosomal location details of *BnBGLU* genes were retrieved through the *B. napus* Multi-Omics Information Resource (BnIR, https://yanglab.hzau.edu.cn/BnIR (accessed on 11 November 2024)) [66]. Subsequently, MG2C v2.1 (http://mg2c.iask.in/mg2c_v2.1/index.html (accessed on 27 January 2025)) was employed to map the *BnBGLU* family genes onto chromosomes [73].

### 4.5. Analysis of the Colinearity of BnBGLU Genes

To comprehensively explore the collinearity relationships, TBtools-II (version 2.315) “One Step MCScanX” module was utilized with default parameters to analyze the homologous relationships among *BnBGLU* genes [74]. Furthermore, the nonsynonymous substitution rates (Ka), synonymous substitution rates (Ks), and Ka/Ks ratios were computed using the default parameters in TBtools-II (version 2.315) “Simple Ka/Ks Calculator” module to assess the selection pressures during evolution [68].

### 4.6. Cis-Regulatory Element Analysis in the Promoter Region of BnBGLU Genes

Cis-regulatory elements within the 2000 bp upstream promoter sequences of *BnBGLUs* were analyzed using the PlantCARE database (version 1; http://bioinformatics.psb.ugent.be/webtools/plantcare/html/ (accessed on 10 May 2025)) and visualized using TBtools-II (version 2.315) “Simple BioSequence Viewer” module [68,75].

### 4.7. Expression Analysis of BnBGLU Genes

To elucidate the functional roles of *BnBGLUs* in abiotic stress adaptation, phytohormone responses, and tissue-specific regulation, transcriptomic datasets under phosphate limitation and phytohormone treatments (precomputed FPKM values) were retrieved from the *B. napus* Genome Annotation Database (BnaGADB v1.0; http://www.bnagadb.cn/ (accessed on 15 May 2025)), while multi-organ expression profiles of the cultivar ZS11 (precomputed FPKM values) were acquired from the *B. napus* Multi-Omics Information Resource (BnIR; https://yanglab.hzau.edu.cn/BnIR (accessed on 15 May 2025)) [66]. Expression patterns were visualized using TBtools-II (version 2.315) “Heat Map” module [68]. All datasets were normalized using log_2_ transformation (FPKM values + 1) prior to analysis.

### 4.8. Transient Transformation of BnBGLU77 in N. benthamiana and qRT-PCR Analysis

Total RNA was extracted from *B. napus* leaves (cultivar Zhongshuang 11) using the EZ-10 DNAaway RNA Mini-Preps Kit (Sangon Biotech, Shanghai, China), followed by reverse transcription with HiScript IV All-in-One RT SuperMix (Vazyme Biotech, Nanjing, Jiangsu, China). The coding sequence of *BnBGLU77* was amplified from ZS11 cDNA by PCR. The purified PCR product was first cloned into the *pEASY*^®^-Blunt Simple Cloning Vector using the *pEASY*^®^-Blunt Simple Cloning Kit (TransGen Biotech, Beijing, China) to generate an entry clone according to the manufacturer’s protocol. Next, the *BnBGLU77* coding sequence was transferred from this entry clone into the overexpression vector pNC-Cam33FC using the Nimble Cloning Kit (NC Biotech, Haikou, Hainan, China). To achieve this, the insert fragment was amplified by PCR from the entry clone using primers designed to generate terminal sequences homologous to the pNC-Cam33FC vector. The resulting PCR product and the vector were then directly combined for in vitro recombination using the Nimble Cloning Mix (according to the kit instructions). Finally, the recombinant mix was transformed into competent *Escherichia coli* Trans1-T1 cells (TransGen Biotech, Beijing, China). Positive clones were selected and verified by sequencing (Primers used in the above steps are listed in Table S5 and the structure of the pNC-Cam33FC is shown in Supplementary Figure S1).

Recombinant and empty vectors were introduced into *Agrobacterium tumefaciens* strain GV3101 (Weidi Biotech, Shanghai, China) and infiltrated into *N. benthamiana* leaves using a needleless syringe. Specifically, for each treated leaf, the right side received the recombinant vector while the left side received the empty vector (control). We injected four healthy leaves using the same method. After 24 h of dark incubation followed by 24 h of light exposure, RNA was extracted from infiltrated tissues for qRT-PCR analysis using the Bio-Rad CFX96 system with *Nb26S* as the internal control (Primers used for qRT-PCR are listed in Table S6) [76]. Relative expression levels were calculated via the 2^−ΔΔCt^ method using three technical replicates per biological sample [77]. The expression levels of *BnBGLU77* in *N. benthamiana* were plotted using GraphPad Prism 10.1.2 (GraphPad Software, Boston, MA, USA) [78]. Statistical significance was determined by two-way ANOVA (* p < 0.05, ** p < 0.01, *** p < 0.001).

### 4.9. Untargeted Metabolomics Analysis of BnBGLU77

Leaf tissues of *N*. *benthamiana* exhibiting the highest *BnBGLU77* overexpression levels in qRT-PCR assays were selected for metabolite extraction. Metabolite profiling was conducted using ultra-performance liquid chromatography coupled with heated electrospray ionization tandem mass spectrometry (UPLC-HESI-MS/MS) [79]. The chromatography conditions matched those reported by Qu et al. [79]. Technical parameters and analytical procedures were consistent with those described in prior publications from our laboratory [80]. Raw data were processed via MS-DIAL software (version 4.60) with three mass databases MoNA, MSMS_Public_EXP_VS17, and MSMS_Public_ExpBioInsilico_VS17 (https://systemsomicslab.github.io/compms/msdial/main.html#MSP (accessed on 4 May 2025)) [81]. MS-DIAL parameter settings aligned with methodologies documented in previously published work [80]. PCA and OPLS-DA were performed using MetaboAnalyst 6.0 (https://www.metaboanalyst.ca/ (accessed on 7 May 2025)) [82]. Features meeting the criteria of VIP > 1, |log_2_(fold change)| > 0.585 (upregulated: >0.585; downregulated: <−0.585), and *p* < 0.05 were defined as differentially abundant metabolites. Volcano plots of significantly differentially abundant metabolites and relative abundance plots of four significantly changed compounds containing β-glucosidic bonds were generated using GraphPad Prism 10.1.2 (GraphPad Software, Boston, MA, USA) [78]. Statistical significance was determined by two-way ANOVA (* p < 0.05, ** p < 0.01, *** p < 0.001). The chemical structures of these metabolites were illustrated using KingDraw 3.0 software (KingDraw, Qingdao, Shandong, China).

## 5. Conclusions

This study provides a systematic characterization of GH1 family *BGLU* genes in *B. napus*. Genome-wide screening identified 149 *BnBGLU* genes, whose encoded proteins were phylogenetically categorized into ten distinct subgroups. Comprehensive analyses including exon–intron structure, conserved motif arrangements, chromosomal localization, and collinearity relationships provide fundamental insights into the organization and evolutionary dynamics of the *BnBGLU* gene family. Bioinformatic analysis of cis-acting elements revealed functional involvement of *BnBGLU* genes in stress response mechanisms. This prediction received experimental validation through observed expression profiles under phosphate deficiency and phytohormone treatments. Moreover, tissue expression profiling established tissue-specific expression patterns for these genes across plant tissues. Functional investigation of BnBGLU77 protein demonstrated its dual enzymatic activity, participating not only in β-glucosidic compound hydrolysis but also potentially facilitating β-glucoside biosynthesis through transglycosylation activity. Hydrolysis-derived compounds, including salicylic acid, gentisic acid, and *trans*-cinnamic acid, along with the biosynthesized fatty acid acyl glycoside have been demonstrated to exert extensive regulatory effects on plant stress responses and metabolic adaptations. Collectively, these findings not only establish a foundation for elucidating the functional roles of *BnBGLU* family members in *B. napus* but also provide molecular insights for developing stress-resistant rapeseed cultivars through targeted genetic strategies.

## Data Availability

All additional datasets supporting the findings of this study are included within the article and Supplementary Materials.

## Supplementary Materials

The following supporting information can be downloaded at: https://www.mdpi.com/article/10.3390/plants14172686/s1, Supplementary Figure S1: Schematic diagram of the plasmid vector pNC-Cam33FC; Supplementary Table S1: The physicochemical properties of BGLU proteins in *B. napus* (ZS11 ecotype); Table S2: Analysis and distribution of conserved motifs in BnBGLU proteins; Table S3: Ka, Ks, and Ka/Ks analysis of *BGLU* genes in *B. napus*; Table S4: Ka, Ks, and Ka/Ks analysis of *BGLU* genes between *A. thaliana* and *B. napus;* Table S5: Primers for amplification of *BnBGLU77* and its Nimble Cloning-ready fragment used in this study; Table S6: Primers used for qRT-PCR analysis in this study.

## References

[1] Mareri L., Parrotta L., Cai G. (2022). Environmental Stress and Plants. Int. J. Mol. Sci..

[2] Wang Y., Mostafa S., Zeng W., Jin B. (2021). Function and Mechanism of Jasmonic Acid in Plant Responses to Abiotic and Biotic Stresses. Int. J. Mol. Sci..

[3] Zhang H., Zhu J., Gong Z., Zhu J.-K. (2022). Abiotic Stress Responses in Plants. Nat. Rev. Genet..

[4] Nawaz M., Sun J., Shabbir S., Khattak W.A., Ren G., Nie X., Bo Y., Javed Q., Du D., Sonne C. (2023). A Review of Plants Strategies to Resist Biotic and Abiotic Environmental Stressors. Sci. Total Environ..

[5] Sato H., Mizoi J., Shinozaki K., Yamaguchi-Shinozaki K. (2024). Complex Plant Responses to Drought and Heat Stress under Climate Change. Plant J. Cell Mol. Biol..

[6] Yang C., Tian F., Ma J., Chen M., Shi X., Chen D., Xie Y., Zhou X., Zhou Z., Dai X. (2023). Glycosylation of Secondary Metabolites: A Multifunctional UDP-Glycosyltransferase, CsUGT74Y1, Promotes the Growth of Plants. J. Agric. Food. Chem..

[7] Tiwari P., Sangwan R.S., Sangwan N.S. (2016). Plant Secondary Metabolism Linked Glycosyltransferases: An Update on Expanding Knowledge and Scopes. Biotechnol. Adv..

[8] Wang X. (2009). Structure, Mechanism and Engineering of Plant Natural Product Glycosyltransferases. FEBS Lett..

[9] Ponnu J., Wahl V., Schmid M. (2011). Trehalose-6-Phosphate: Connecting Plant Metabolism and Development. Front. Plant Sci..

[10] Lu M., Zhao Y., Feng Y., Tang X., Zhao W., Yu K., Pan Y., Wang Q., Cui J., Zhang M. (2023). 2,4-dihydroxybenzoic Acid, a Novel SA Derivative, Controls Plant Immunity via UGT95B17-mediated Glucosylation: A Case Study in *Camellia sinensis*. Adv. Sci..

[11] Lamaoui M., Jemo M., Datla R., Bekkaoui F. (2018). Heat and Drought Stresses in Crops and Approaches for Their Mitigation. Front. Chem..

[12] Huang S., Zhang S., Ma X., Zheng X., Liu Y., Zhu Q., Luo X., Cui J., Song C. (2025). Glycoside-Specific Metabolomics Reveals the Novel Mechanism of Glycinebetaine-Induced Cold Tolerance by Regulating Apigenin Glycosylation in Tea Plants. New Phytol..

[13] Chandrasekar B., Colby T., Emran Khan Emon A., Jiang J., Hong T.N., Villamor J.G., Harzen A., Overkleeft H.S., van der Hoorn R.A.L. (2014). Broad-Range Glycosidase Activity Profiling. Mol. Cell. Proteom.

[14] Kong H., Song J., Ma S., Yang J., Shao Z., Li Q., Li Z., Xie Z., Yang P., Cao Y. (2024). Genome-Wide Identification and Expression Analysis of the Glycosyl Hydrolase Family 1 Genes in Medicago Sativa Revealed Their Potential Roles in Response to Multiple Abiotic Stresses. BMC Genom..

[15] Chuenchor W., Pengthaisong S., Robinson R.C., Yuvaniyama J., Oonanant W., Bevan D.R., Esen A., Chen C.-J., Opassiri R., Svasti J. (2008). Structural Insights into Rice BGlu1 Beta-Glucosidase Oligosaccharide Hydrolysis and Transglycosylation. J. Mol. Biol..

[16] Baba S.A., Vishwakarma R.A., Ashraf N. (2017). Functional Characterization of CsBGlu12, a β-Glucosidase from *Crocus sativus*, Provides Insights into Its Role in Abiotic Stress through Accumulation of Antioxidant Flavonols. J. Biol. Chem..

[17] Ouyang B., Wang G., Zhang N., Zuo J., Huang Y., Zhao X. (2023). Recent Advances in β-Glucosidase Sequence and Structure Engineering: A Brief Review. Molecules.

[18] Chen H., Jin X., Zhu L., Lu Y., Ma Z., Liu S., Chen X. (2020). Glycosyl Hydrolase Catalyzed Glycosylation in Unconventional Media. Appl. Microbiol. Biotechnol..

[19] Godse R., Bawane H., Tripathi J., Kulkarni R. (2021). Unconventional β-Glucosidases: A Promising Biocatalyst for Industrial Biotechnology. Appl. Biochem. Biotechnol..

[20] Bednarek P., Pislewska-Bednarek M., Svatos A., Schneider B., Doubsky J., Mansurova M., Humphry M., Consonni C., Panstruga R., Sanchez-Vallet A. (2009). A Glucosinolate Metabolism Pathway in Living Plant Cells Mediates Broad-Spectrum Antifungal Defense. Science.

[21] Zamioudis C., Hanson J., Pieterse C.M.J. (2014). β-Glucosidase BGLU42 Is a MYB72-Dependent Key Regulator of Rhizobacteria-Induced Systemic Resistance and Modulates Iron Deficiency Responses in Arabidopsis Roots. New Phytol..

[22] Ren R., Li D., Zhen C., Chen D., Chen X. (2019). Specific Roles of Os4BGlu10, Os6BGlu24, and Os9BGlu33 in Seed Germination, Root Elongation, and Drought Tolerance in Rice. Planta.

[23] Yang H., Yao X., Wu W., He A., Ma C., Yang S., Ruan J. (2024). Genome-Wide Identification and Gene Expression Pattern Analysis of the Glycoside Hydrolase Family 1 in *Fagopyrum tataricum*. BMC Plant Biol..

[24] Frommann J.-F., Pucker B., Sielmann L.M., Müller C., Weisshaar B., Stracke R., Schweiger R. (2025). Metabolic Fingerprinting Reveals Roles of *Arabidopsis thaliana* BGLU1, BGLU3, and BGLU4 in Glycosylation of Various Flavonoids. Phytochemistry.

[25] Wu J., Lv S., Zhao L., Gao T., Yu C., Hu J., Ma F. (2023). Advances in the Study of the Function and Mechanism of the Action of Flavonoids in Plants under Environmental Stresses. Planta.

[26] Mohnen D., Hahn M.G. (1993). Cell Wall Carbohydrates as Signals in Plants. Semin. Cell. Biol..

[27] Chen Y.-Y., Lu H.-Q., Jiang K.-X., Wang Y.-R., Wang Y.-P., Jiang J.-J. (2022). The Flavonoid Biosynthesis and Regulation in *Brassica napus*: A Review. Int. J. Mol. Sci..

[28] Gómez-Anduro G., Ceniceros-Ojeda E.A., Casados-Vázquez L.E., Bencivenni C., Sierra-Beltrán A., Murillo-Amador B., Tiessen A. (2011). Genome-Wide Analysis of the Beta-Glucosidase Gene Family in Maize (*Zea mays* L. Var B73). Plant Mol. Biol..

[29] Wang H., Zhang Y., Feng X., Peng F., Mazoor M.A., Zhang Y., Zhao Y., Han W., Lu J., Cao Y. (2022). Analysis of the β-Glucosidase Family Reveals Genes Involved in the Lignification of Stone Cells in Chinese White Pear (*Pyrus bretschneideri* Rehd.). Front. Plant Sci..

[30] Thorlby G., Fourrier N., Warren G. (2004). The SENSITIVE TO FREEZING2 Gene, Required for Freezing Tolerance in *Arabidopsis thaliana*, Encodes a Beta-Glucosidase. Plant Cell.

[31] Ishihara H., Tohge T., Viehöver P., Fernie A.R., Weisshaar B., Stracke R. (2016). Natural Variation in Flavonol Accumulation in Arabidopsis Is Determined by the Flavonol Glucosyltransferase BGLU6. J. Exp. Bot..

[32] Escamilla-Treviño L.L., Chen W., Card M.L., Shih M.-C., Cheng C.-L., Poulton J.E. (2006). *Arabidopsis thaliana* Beta-Glucosidases BGLU45 and BGLU46 Hydrolyse Monolignol Glucosides. Phytochemistry.

[33] Chapelle A., Morreel K., Vanholme R., Le-Bris P., Morin H., Lapierre C., Boerjan W., Jouanin L., Demont-Caulet N. (2012). Impact of the Absence of Stem-Specific β-Glucosidases on Lignin and Monolignols. Plant Physiol..

[34] Xu Z., Escamilla-Treviño L., Zeng L., Lalgondar M., Bevan D., Winkel B., Mohamed A., Cheng C.-L., Shih M.-C., Poulton J. (2004). Functional Genomic Analysis of *Arabidopsis thaliana* Glycoside Hydrolase Family 1. Plant Mol. Biol..

[35] Burmeister W.P., Cottaz S., Driguez H., Iori R., Palmieri S., Henrissat B. (1997). The Crystal Structures of *Sinapis alba* Myrosinase and a Covalent Glycosyl-Enzyme Intermediate Provide Insights into the Substrate Recognition and Active-Site Machinery of an S-Glycosidase. Structure.

[36] Qiao X., Yin H., Li L., Wang R., Wu J., Wu J., Zhang S. (2018). Different Modes of Gene Duplication Show Divergent Evolutionary Patterns and Contribute Differently to the Expansion of Gene Families Involved in Important Fruit Traits in Pear (*Pyrus bretschneideri*). Front. Plant Sci..

[37] Panchy N., Lehti-Shiu M., Shiu S.-H. (2016). Evolution of Gene Duplication in Plants1[OPEN]. Plant Physiol..

[38] Roth C., Liberles D.A. (2006). A Systematic Search for Positive Selection in Higher Plants (Embryophytes). BMC Plant Biol..

[39] Brunet T.D.P., Doolittle W.F., Bielawski J.P. (2021). The Role of Purifying Selection in the Origin and Maintenance of Complex Function. Stud. Hist. Philos. Sci..

[40] Hurst L.D. (2002). The Ka/Ks Ratio: Diagnosing the Form of Sequence Evolution. Trends Genet. TIG.

[41] Halder K., Chaudhuri A., Abdin M.Z., Majee M., Datta A. (2022). Chromatin-Based Transcriptional Reprogramming in Plants under Abiotic Stresses. Plants.

[42] Richardson A. (2020). Plant Development: Coordinating across Space and Time. Curr. Biol. CB.

[43] Niu Y.F., Chai R.S., Jin G.L., Wang H., Tang C.X., Zhang Y.S. (2013). Responses of Root Architecture Development to Low Phosphorus Availability: A Review. Ann. Bot..

[44] Malboobi M.A., Lefebvre D.D. (1997). A Phosphate-Starvation Inducible Beta-Glucosidase Gene (Psr3.2) Isolated from *Arabidopsis thaliana* Is a Member of a Distinct Subfamily of the BGA Family. Plant Mol. Biol..

[45] Hammond J.P., Bennett M.J., Bowen H.C., Broadley M.R., Eastwood D.C., May S.T., Rahn C., Swarup R., Woolaway K.E., White P.J. (2003). Changes in Gene Expression in Arabidopsis Shoots during Phosphate Starvation and the Potential for Developing Smart Plants. Plant Physiol..

[46] Zhang J., Xu L., Wang F., Deng M., Yi K. (2012). Modulating the Root Elongation by Phosphate/Nitrogen Starvation in an OsGLU3 Dependant Way in Rice. Plant Signal. Behav..

[47] Waadt R., Seller C.A., Hsu P.-K., Takahashi Y., Munemasa S., Schroeder J.I. (2022). Plant Hormone Regulation of Abiotic Stress Responses. Nat. Rev. Mol. Cell Biol..

[48] Romero-Téllez S., Lluch J.M., González-Lafont À., Masgrau L. (2019). Comparing Hydrolysis and Transglycosylation Reactions Catalyzed by *Thermus thermophilus* β-Glycosidase. A Combined MD and QM/MM Study. Front. Chem..

[49] Grellet Bournonville C., Filippone M.P., Di Peto P.d.L.Á., Trejo M.F., Couto A.S., Mamaní de Marchese A., Díaz Ricci J.C., Welin B., Castagnaro A.P. (2020). Strawberry Fatty Acyl Glycosides Enhance Disease Protection, Have Antibiotic Activity and Stimulate Plant Growth. Sci. Rep..

[50] Mutschler M.A., Kennedy G.G., Ullman D.E. (2023). Acylsugar-Mediated Resistance as Part of a Multilayered Defense against Thrips, Orthotospoviruses, and Beyond. Curr. Opin. Insect Sci..

[51] Chen J., Clinton M., Qi G., Wang D., Liu F., Fu Z.Q. (2020). Reprogramming and Remodeling: Transcriptional and Epigenetic Regulation of Salicylic Acid-Mediated Plant Defense. J. Exp. Bot..

[52] Koo Y.M., Heo A.Y., Choi H.W. (2020). Salicylic Acid as a Safe Plant Protector and Growth Regulator. Plant Pathol. J..

[53] Bellés J.M., Garro R., Pallás V., Fayos J., Rodrigo I., Conejero V. (2006). Accumulation of Gentisic Acid as Associated with Systemic Infections but Not with the Hypersensitive Response in Plant-Pathogen Interactions. Planta.

[54] Fayos J., Bellés J.M., López-Gresa M.P., Primo J., Conejero V. (2006). Induction of Gentisic Acid 5-O-Beta-D-Xylopyranoside in Tomato and Cucumber Plants Infected by Different Pathogens. Phytochemistry.

[55] López-González D., Bruno L., Díaz-Tielas C., Lupini A., Aci M.M., Talarico E., Madeo M.L., Muto A., Sánchez-Moreiras A.M., Araniti F. (2023). Short-Term Effects of Trans-Cinnamic Acid on the Metabolism of *Zea mays* L. Roots. Plants.

[56] Moreno-Robles A., Cala Peralta A., Zorrilla J.G., Soriano G., Masi M., Vilariño-Rodríguez S., Cimmino A., Fernández-Aparicio M. (2023). Structure-Activity Relationship (SAR) Study of Trans-Cinnamic Acid and Derivatives on the Parasitic Weed *Cuscuta campestris*. Plants.

[57] Li Z., Liu H., Ding Z., Yan J., Yu H., Pan R., Hu J., Guan Y., Hua J. (2020). Low Temperature Enhances Plant Immunity via Salicylic Acid Pathway Genes That Are Repressed by Ethylene. Plant Physiol..

[58] Liang B., Wang H., Yang C., Wang L., Qi L., Guo Z., Chen X. (2022). Salicylic Acid Is Required for Broad-Spectrum Disease Resistance in Rice. Int. J. Mol. Sci..

[59] Vlaminck L., De Rouck B., Desmet S., Van Gerrewey T., Goeminne G., De Smet L., Storme V., Kyndt T., Demeestere K., Gheysen G. (2022). Opposing Effects of Trans- and Cis-Cinnamic Acid during Rice Coleoptile Elongation. Plant Direct.

[60] Remali J., Sahidin I., Aizat W.M. (2022). Xanthone Biosynthetic Pathway in Plants: A Review. Front. Plant Sci..

[61] Xiang L., Etxeberria E., Van den Ende W. (2013). Vacuolar Protein Sorting Mechanisms in Plants. FEBS J..

[62] Vaschetto L.M., Ortiz N. (2019). The Role of Sequence Duplication in Transcriptional Regulation and Genome Evolution. Curr. Genom..

[63] De la Concepcion J.C., Vega Benjumea J., Bialas A., Terauchi R., Kamoun S., Banfield M.J. (2021). Functional Diversification Gave Rise to Allelic Specialization in a Rice NLR Immune Receptor Pair. eLife.

[64] Gelfman S., Burstein D., Penn O., Savchenko A., Amit M., Schwartz S., Pupko T., Ast G. (2012). Changes in Exon-Intron Structure during Vertebrate Evolution Affect the Splicing Pattern of Exons. Genome Res..

[65] Reiser L., Subramaniam S., Zhang P., Berardini T. (2022). Using the Arabidopsis Information Resource (TAIR) to Find Information about Arabidopsis Genes. Curr. Protocol..

[66] Yang Z., Wang S., Wei L., Huang Y., Liu D., Jia Y., Luo C., Lin Y., Liang C., Hu Y. (2023). BnIR: A Multi-Omics Database with Various Tools for *Brassica napus* Research and Breeding. Mol. Plant.

[67] Mistry J., Chuguransky S., Williams L., Qureshi M., Salazar G.A., Sonnhammer E.L.L., Tosatto S.C.E., Paladin L., Raj S., Richardson L.J. (2021). Pfam: The Protein Families Database in 2021. Nucleic Acids Res..

[68] Chen C., Wu Y., Li J., Wang X., Zeng Z., Xu J., Liu Y., Feng J., Chen H., He Y. (2023). TBtools-II: A “One for All, All for One” Bioinformatics Platform for Biological Big-Data Mining. Mol. Plant.

[69] Yang M., Derbyshire M.K., Yamashita R.A., Marchler-Bauer A. (2020). NCBI’s Conserved Domain Database and Tools for Protein Domain Analysis. Curr. Protoc. Bioinform..

[70] Chou K.-C., Shen H.-B. (2008). Cell-PLoc: A Package of Web Servers for Predicting Subcellular Localization of Proteins in Various Organisms. Nat. Protoc..

[71] Letunic I., Bork P. (2024). Interactive Tree of Life (iTOL) v6: Recent Updates to the Phylogenetic Tree Display and Annotation Tool. Nucleic Acids Res..

[72] Bailey T.L., Boden M., Buske F.A., Frith M., Grant C.E., Clementi L., Ren J., Li W.W., Noble W.S. (2009). MEME SUITE: Tools for Motif Discovery and Searching. Nucleic Acids Res..

[73] Chao J., Li Z., Sun Y., Aluko O.O., Wu X., Wang Q., Liu G. (2021). MG2C: A User-Friendly Online Tool for Drawing Genetic Maps. Mol. Hortic..

[74] Wang Y., Tang H., Debarry J.D., Tan X., Li J., Wang X., Lee T., Jin H., Marler B., Guo H. (2012). MCScanX: A Toolkit for Detection and Evolutionary Analysis of Gene Synteny and Collinearity. Nucleic Acids Res..

[75] Lescot M., Déhais P., Thijs G., Marchal K., Moreau Y., Van de Peer Y., Rouzé P., Rombauts S. (2002). PlantCARE, a Database of Plant Cis-Acting Regulatory Elements and a Portal to Tools for in Silico Analysis of Promoter Sequences. Nucleic Acids Res..

[76] Dong C., Qu G., Guo J., Wei F., Gao S., Sun Z., Jin L., Sun X., Rochaix J.-D., Miao Y. (2022). Rational Design of Geranylgeranyl Diphosphate Synthase Enhances Carotenoid Production and Improves Photosynthetic Efficiency in Nicotiana Tabacum. Sci. Bull..

[77] Wu G., Zhang L., Wu Y., Cao Y., Lu C. (2010). Comparison of Five Endogenous Reference Genes for Specific PCR Detection and Quantification of *Brassica napus*. J. Agric. Food Chem..

[78] Mitteer D.R., Greer B.D. (2022). Using GraphPad Prism’s Heat Maps for Efficient, Fine-Grained Analyses of Single-Case Data. Behav. Anal. Pract..

[79] Qu C., Yin N., Chen S., Wang S., Chen X., Zhao H., Shen S., Fu F., Zhou B., Xu X. (2020). Comparative Analysis of the Metabolic Profiles of Yellow- versus Black-Seeded Rapeseed Using UPLC-HESI-MS/MS and Transcriptome Analysis. J. Agric. Food Chem..

[80] Shen S., Tang Y., Liu D., Chen L., Zhang Y., Ye K., Sun F., Wei X., Du H., Zhao H. (2025). Untargeted Metabolomics Analysis Reveals Differential Accumulation of Flavonoids between Yellow-Seeded and Black-Seeded Rapeseed Varieties. Plants.

[81] Tsugawa H., Ikeda K., Takahashi M., Satoh A., Mori Y., Uchino H., Okahashi N., Yamada Y., Tada I., Bonini P. (2020). A Lipidome Atlas in MS-DIAL 4. Nat. Biotechnol..

[82] Pang Z., Lu Y., Zhou G., Hui F., Xu L., Viau C., Spigelman A.F., MacDonald P.E., Wishart D.S., Li S. (2024). MetaboAnalyst 6.0: Towards a Unified Platform for Metabolomics Data Processing, Analysis and Interpretation. Nucleic Acids Res..

