# Supplementary figures and images for: Genome-Wide Identification of the Glycosyl Hydrolase Family 1 Genes in Brassica napus L. and Functional Characterization of BnBGLU77
Source: Plants (Basel). 2025 Aug 28;14(17):2686. doi: 10.3390/plants14172686 (PMC12430181; doi:10.3390/plants14172686)

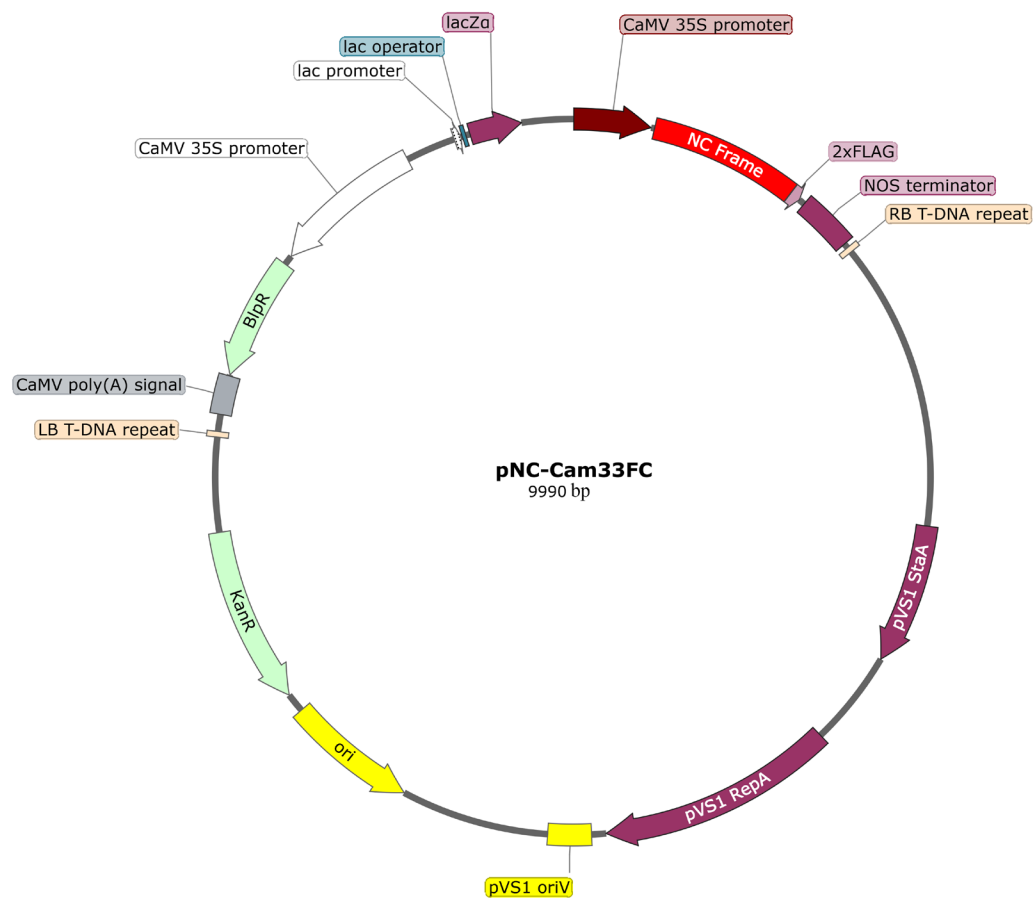

**Supplementary Figure S1.** Schematic diagram of the plasmid vector pNC-Cam33FC.

Supplement: Supplementary file 1 [file plants-14-02686-s001.zip › Supplementary Figure.pdf]
